# Neuron Biomechanics Probed by Atomic Force Microscopy

**DOI:** 10.3390/ijms140816124

**Published:** 2013-08-05

**Authors:** Elise Spedden, Cristian Staii

**Affiliations:** Department of Physics and Astronomy and Center for Nanoscopic Physics, Tufts University, 4 Colby Street, Medford, MA 02155, USA; E-Mail: elise.spedden@tufts.edu

**Keywords:** Atomic Force Microscopy, neurons, cellular elasticity, cellular biomechanics, cytoskeletal dynamics

## Abstract

Mechanical interactions play a key role in many processes associated with neuronal growth and development. Over the last few years there has been significant progress in our understanding of the role played by the substrate stiffness in neuronal growth, of the cell-substrate adhesion forces, of the generation of traction forces during axonal elongation, and of the relationships between the neuron soma elastic properties and its health. The particular capabilities of the Atomic Force Microscope (AFM), such as high spatial resolution, high degree of control over the magnitude and orientation of the applied forces, minimal sample damage, and the ability to image and interact with cells in physiologically relevant conditions make this technique particularly suitable for measuring mechanical properties of living neuronal cells. This article reviews recent advances on using the AFM for studying neuronal biomechanics, provides an overview about the state-of-the-art measurements, and suggests directions for future applications.

## 1. Introduction

Neurons are highly specialized cells primarily responsible for transmitting information through chemical and electrical signaling in both the central and peripheral nervous system. They are composed of a cell body (soma), as well as dendrites and axons (neurites) which extend from the soma. The structural and mechanical properties of living neurons are essential components that govern many neuronal growth and regeneration processes including axonal extension, generation of traction forces and interactions between neurons and the surrounding environment such as the growth substrate, extracellular matrix, glial cells or other neurons [1]. The structural and mechanical properties of neurons are influenced by cytoskeletal components (microtubules and actin filaments), the cytoplasm and cell nucleus, and by the coupling between neurons and the extracellular matrix, which is involved in cell-substrate adhesion, and provides the anchoring needed for processes such as axonal extension and cell migration [2]. Recent advances in micro-spectroscopy techniques have allowed for the study of the structural and mechanical properties of living cells at increasing resolution. The Atomic Force Microscope (AFM) is at the forefront of this exploration. The AFM is a unique tool which enables researchers to obtain high resolution topographical data, control forces applied to cells, measure cellular elastic properties, and monitor variations in elastic modulus across living cells. Controlled forces have also been used to precisely manipulate the cells under study.

Excellent review papers are available concerning a wide array of topics related to the brain and nervous system, as well as AFM study of many types of living cells. These range from broader reviews of nervous system mechanics, mechanosensitivty and growth [1,3], to more specific reviews of AFM applications in biology [4–10]. Here, we present a focused and comprehensive review of the use of the AFM for the direct study of individual neuronal cells. We cover recent advancements in AFM-based techniques for neuronal measurement and summarize their results. We also describe in detail AFM measurements of neuronal mechanics, and how the measured elastic properties vary across different neuronal cell types, and between different areas of the same cell.

## 2. Cellular Topography and Elastic Properties Measured by AFM

AFM measurement relies on the motions of a sharp tip positioned at the end of a flexible cantilever, which interacts with the sample via surface forces such as Van der Waals forces, electrostatic forces and capillary forces [5,11]. The cantilever behaves like a spring, so that any forces acting on the AFM tip cause the cantilever to deflect. These interactions are detected by the positioning of a laser reflected off of the back of the cantilever onto a 4-quadrant photo sensor.

### 2.1. Modes of AFM Measurement

**Figure 1:**
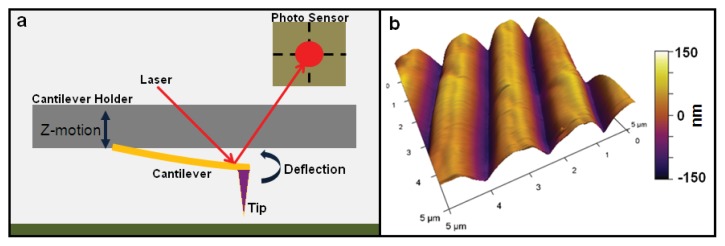
(**a**) Schematic of Atomic Force Microscope (AFM) cantilever holder and laser detector setup. Before calibration, tip deflection is measured as a voltage value generated by a difference in light falling on the upper *vs.* lower quadrants of the photo sensor. In order to measure cantilever deflection as a distance, the cantilever must be calibrated. This is typically done by performing a force curve on an “infinitely” hard surface (zero indentation), and equating the decrease in cantilever z height after contact with the increase in tip deflection [5]; (**b**) AFM topography image of ridges on a micropatterned silicon substrate shown as an example of a representative AFM image. Image taken by Elise Spedden in Staii lab at Tufts University.

Several modes of topographical measurement are possible. Here we focus on contact mode and tapping mode, which are the two most commonly used for measuring cells. In basic contact mode scanning, topography is determined by a feedback mechanism which maintains a constant cantilever deflection against the sample surface. This is done by moving the z position of the cantilever up and down in response to changes in deflection (Figure 1a). This maintains a constant force on the surface during scanning. Another widely used imaging mode is intermittent contact (AC), or “tapping” mode, where the cantilever is driven to oscillate near its resonant frequency, and surface forces are measured by the damping of these oscillations [5]. In this mode the cantilever z position is adjusted to maintain a constant set-point oscillation amplitude of the cantilever, and its vertical position at each point forms the topographical image of the sample. For both contact and tapping modes, adjustments in the z position of the cantilever yield the sample height, so the actual values for deflection or oscillation amplitude are not important for acquiring the sample topography. For elastic modulus measurements, however, both the actual cantilever deflection distance, and the cantilever spring constant are needed to determine cantilever force and indentation values, as described below.

**Figure 2:**
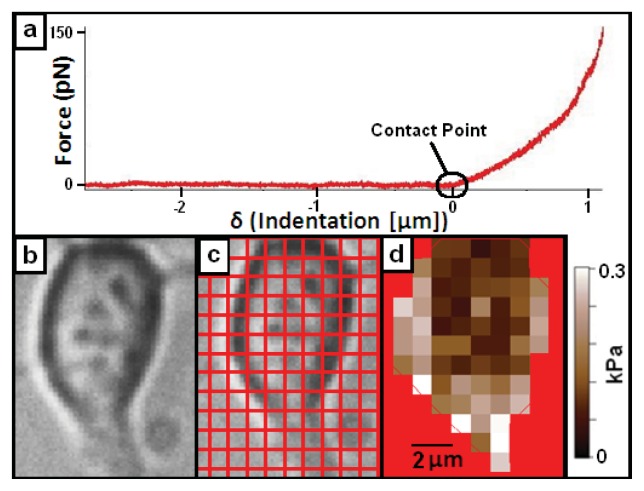
(**a**) Typical AFM force *vs.* indentation curve. The contact point is defined as the point at which the force *vs.* indentation slope begins to increase above noise level. The flat line indicates the approach portion, where the cantilever is moving down but it is not yet in contact with the surface. The curved portion after the contact point indicates how the total force of the tip on the sample changes as the cantilever indents the surface; (**b**) Optical image of living cortical neuron; (**c**) Neuron shown in (**a**) with superimposed 1 × 1 micron grid; (**d**) Elastic modulus map of neuron from (**c**), where force *vs.* indentation curves are taken at each point on the grid and elastic modulus values are calculated from the indentation curves (see text). These images illustrate the elasticity mapping procedure where optical and AFM measurements are taken simultaneously. Images taken by Elise Spedden, in Staii lab at Tufts University.

**Figure 3:**
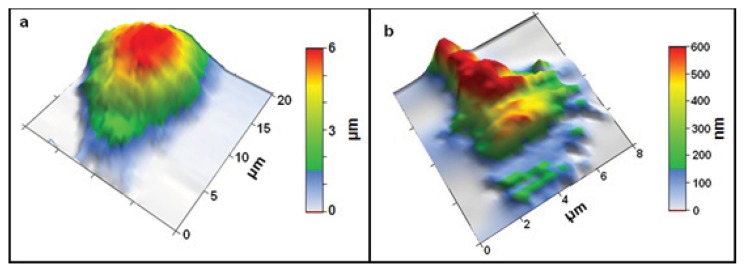
(**a**) Example of a 3D AFM topography image of a live cortical neuron soma; (**b**) 3D AFM topography image of a live neuron growth cone. Both images were taken in force-volume mode, thus rendering lower spatial resolution than high-resolution AFM topography in tapping mode. However, force-volume mode images contain both topographical and elasticity information as explained in the main text. Images taken by Elise Spedden in Staii lab at Tufts University.

Determining the elastic modulus of a cell via AFM typically involves taking force *vs.* indentation curves on the sample (Figure 2a). A force curve will move the z position of the cantilever down until the cantilever is deflected a set amount (the trigger value) and then retracted. The elastic modulus of the sample can be determined by fitting the force *vs.* indentation curve above the contact point [6,9]. Indentation is obtained by subtracting cantilever upward deflection from the downward z-motion of the AFM head, and force is obtained from the deflection value by treating the cantilever as a spring obeying Hooke’s law. These force *vs.* indentation curves can be measured on specific locations on a living or fixed sample, or they can be taken in a grid pattern across the sample surface (Figure 2b–d). This allows maps of elastic modulus values to be obtained across a sample [12]. This technique is sometimes referred to as force-volume mode as these maps also contain simultaneous topographical information over the cells which can be used to model the cells in 3 dimensions (see Figure 3).

### 2.2. The Hertz Model Applied to AFM

The elastic modulus of a material (Young’s modulus) is defined as the ratio between the uniaxial stress (force) applied to the material and the uniaxial strain (deformation) that it undergoes. The Hertz model, as applied to AFM relates the total cantilever force and maximum indentation into the sample to the sample elastic modulus [6,9]. The specific relationship varies for different cantilever tip geometries. Most common for AFM applications are the models for the sphere and cone shaped tips [9]. There are also other tip shapes, such as 3 and 4-sided pyramids, although those tip shapes are often approximated as conical [10]. Conical or pyramidal shaped tips are best for high resolution topographical scanning, as well as for mapping elastic modulus values with high spatial resolution. Spherical tips have the advantage of distributing the applied forces over larger areas. This is useful for limiting any potential damage to the cell (which is an important concern if high forces are used), and for obtaining averaged elastic modulus values of a whole cell soma or entire tissue areas. The Hertz model as applied to AFM requires knowledge of the tip geometry. In the case of the spherical tip, this means the radius of sphere used (*R*). In the case of conical indenters the relevant quantity is the half-angle of the conical indenter (α). These values relate to how the tip-sample contact area and forces are expected to change during indentation. The Hertz model as applied to AFM for a spherical indenter is given in Equation 1, and for a conical indenter in Equation 2 [9].

(1) $$F=\frac{4}{3}\frac{E}{\left(1-\nu^{2}\right)}\sqrt{R}\delta^{\frac{3}{2}}$$

(2) $$F=\frac{E}{\left(1-\nu^{2}\right)}\frac{2\;\text{tan}(\alpha )}{\pi }\delta^{2}$$

In these equations *F* is the force, *E* is the elastic modulus, and δ is the indentation depth. Also important in these equations is a value known as the Poisson ratio (*ν*). This value relates to the samples ability to “bulge out” when compressed. For example, in the case of a rubber ball, the ball expands laterally as it is being compressed from above, maintaining a constant volume. This redistributes some of the material strain in the lateral direction. Cork, on the other hand, typically compresses without bulging. In general, it is important to know for any material how much of the stress on the system goes into uniaxial strain in the direction of pressure, and how much is distributed into lateral strains. This relationship between strains parallel and perpendicular to the stress on a system is called the Poisson ratio of the material. The bulging rubber ball exemplifies a Poisson ratio close to 0.5, whereas the fully compressible cork would be closer to 0. For living cells the value is typically chosen as being between 0.3 and 0.5 representing a mostly or fully incompressible system. While most cell types have not been measured for their Poisson ratio, a value of 0.5 is often chosen, assuming that typical living cells are fully incompressible [4]. However, several types of cells that have been measured yield lower values for their Poisson ratio. For example, a value of 0.38 has been measured for chondrocytes [13].

While the Hertz model is currently by far the most common method of elastic modulus evaluation of force curves from living cells, the model makes several assumptions. These assumptions result in limitations both on what can be measured using this method and how to compare the results with other measurements obtained using different models.

First, the Hertz model assumes that the material is behaving elastically and will return to its initial state after the force is removed. To an extent, the validity of this assumption for any given sample can be checked by the shape and repeatability of the force curves acquired. Though cells do not behave exactly like perfect elastic materials, the better this condition is met, the more valid the model for the sample in question. Secondly, the Hertz model assumes that the materials are homogeneous and isotropic. This is typically true for the AFM indenters, but it is only an approximation for living cells. In the measurement of living cells multiple layers of inhomogeneous material are being indented together. When the traditional AFM adaptation of the Hertz model is applied to this situation, the “true” elastic modulus values of all materials being intended contribute to the final value measured. This can give a reasonable and consistent measurement of the cell’s elastic response to external forces, but does not give specific information about the individual components being measured. Models have been proposed which isolate elastic modulus information from various portions of a force *vs.* indentation curve to accommodate the multiple-layers encountered by the AFM tip [14,15]. This type of model may be preferred by researchers interested in isolating the individual contributions within multi-layer systems to AFM-acquired force *vs.* indentation curves. A third consideration in this model is the assumption of measurement on a flat surface. As such, researchers must expect additional error in the application of the typical AFM Hertz model for curves near the very edges of a highly curved sample (such as a living soma). These regions also may experience problems such as lateral movement of the sample during curve acquisition. Special care must be taken in considering the validity of curves taken in these areas [12].

## 3. AFM Topography of Neurons

AFM topography is used to image fixed and living neurons at high resolution [16–27]. Many topographical studies fix the neurons prior to measurement [16,17,25,26]. This can combat damage to the cell structure or removal of the cell from the surface due to forces exerted by the AFM tip. AFM topography of fixed hippocampal neurons has provided insight into 3-dimensional (3D) cell structure and location of the cell nucleus, mitochondria, and filaments [25]. This early study was among the first to visualize high resolution neuronal structure in 3D. Fixed growth cones of neurons have been imaged in detail [16] and AFM topography of fixed hippocampal somas, neurites and growth cones can also be utilized to track growth and detailed changes in morphology along patterned substrates [17,26]. The AFM has been used to perform high resolution analysis of axon morphology and height-to-width ratio over time on micropatterned substrates [26]. Topography of fixed growth cones at various stages of growth has presented a decreasing height-to-width ratio as axon development progressed, as well as substantial increase in growth cone flattening over the adhesion molecule L1 as compared to Poly-d-Lysine (PDL). This increase in flattening produced a larger growth cone area and was correlated with highly dynamic outgrowth. This result indicates that an increase in sensing and progression accompanies an increase in growth cone spreading [26].

AFM topography of both fixed and living cells has been obtained for Dorsal Root Ganglia (DRG) neurons, embryonic stem cell-derived neurons, and chick embryo spinal cord neurons [18–23,27]. Nanoscale structural details in fixed growth cones, including low-height regions of the growth cone with an area of 0.01–3.5 μm^2^ and ranging from 2 to 180 nm deep were discovered through AFM topography. These measurements show the significance of the structural properties of actin and tubulin in the growth cone, as the low regions were present primarily in areas devoid of actin or tubulin concentrations. These structures were then confirmed by measurements on living DRGs [21], and later, similar structures were found in the growth cones of living and fixed embryonic stem cell derived neurons [22]. Fluorescence has been combined with AFM to track morphological changes and the destruction of the DRG cytoskeleton upon chemical modification [20]. The effects of acrolein on neuronal morphology have also been studied with the AFM. The study showed that increased presence of acrolein (an aldehyde produced during traumatic brain injury) is a likely contributor to secondary injury after an initial traumatic injury as it produced significant degradation of cytoskeletal structures and a major decrease in cell viability [20]. In a different set of experiments, the AFM tip has been used as an intentional source of neuronal damage prior to imaging. In these studies, living DRG somas and axons were intentionally sliced with the AFM and the cell volume and morphology tracked over time after damage [18]. This use of the AFM yielded unprecedented precision in the study of neuronal injury. Microscale slices in the neuronal soma membrane produced cell death and elimination of cytoplasm, while identical injury to the membrane of neurites did not lead to their destruction. This indicates a higher susceptibility of the soma to injury of this type than of neurites. This study also used AFM to resolve new dynamic architectures in the growth cone at a scale inaccessible from traditional optical microscopy [18].

Live hippocampal neurons are not typically imaged using traditional tapping or contact mode in fluid due to their extremely soft and malleable nature. Magnetic AC (MAC) mode, a modification of tapping mode where a specialized AFM cantilever is driven at its tip via an external magnetic field, has been used to image the topography of living hippocampal neurons [24]. Driving the cantilever at the tip rather than the base minimizes changes in the tip drive amplitude and phase due to the surrounding fluid and allows for a more precise measurement of changes in cantilever oscillation due to surface interactions. This also allows clearer images to be taken in fluid at a lower scanning force. This low-force imaging allowed the researchers to monitor cellular damage. Neural spine damage was observed under moderate force, and MAC imaging was used to track neuronal regeneration. Neurons were also chemically modified with Aβ25–35 to determine its effects on growing neurons. This chemical is thought to be involved in the initiation of Alzheimer’s disease. Changes induced by this modification were studied via MAC mode topography scanning and included the gradual contraction and obliteration of the growth cone of previously growing axons [24].

AFM topography can be used to monitor changes in living neurons over time, and is a valuable tool for high resolution detection and monitoring of neuronal morphology. Limitations of this technique include the necessity for neurons to be well adhered to the sample, to be confined to 2-dimensional substrates, and to change their morphology over time scales which are slower than the typical time necessary to acquire the AFM image (1–5 min). These constrains are particularly important when imaging active growth cones [23].

## 4. Mechanical Properties of Neurons

The AFM is an ideal tool for micromechanical measurements. AFM has been used to determine elastic modulus values on live and fixed neurons, as well as slices and explants from the brain (see Table 1). Techniques for micromechanical measurements vary. Spherical AFM tips are common for tissue or bulk soma measurements [28–32]. Smaller cone or pyramidal AFM tips are typically used for measurements on particular points across the cell or for mapping the elastic modulus over entire regions [12,27,33,34].

### 4.1. Fixed Neurons

Fixing neuronal cells prior to elastic modulus measurement helps circumvent many potential issues that accompany live measurement such as problems with cell-substrate adhesion, cell motility and the maintenance of neuronal viability during measurement. Fixation also may allow for the imaging of structures which are otherwise too dynamic to capture on the timescales required for AFM scanning. On fixed neuronal cells mechanical measurements have been performed on soma and growth cone regions. Fixed neurons typically yield elastic modulus values on the order of 10 to hundreds of kPa [33,35]. These values depend on both the type of neuron, and region of cell measured. Fixed primary spinal cord neurons measured at various locations on the soma with a pyramidal tip yield elastic modulus values of the order of 10 kPa [33]. This study aimed to determine if the elastic modulus of neurons responds to changes in substrate stiffness, as other cell types, such as fibroblasts [36], have been shown to do. It was found that fixed neurons do show a dependence of the cell elastic modulus on the plating substrate; plating on stiffer polyacrylamide gels yields stiffer elastic modulus values over the fixed cell soma [33]. These results have led to the hypothesis that differences in the production of extracellular matrix between cells on different substrates may account for some of the differences in elastic modulus. Other cell types which exhibit this trend, such as fibroblasts, exhibit changes in cell spreading and stress fiber formations which contribute to the observed differences in the elastic modulus [36]. The above results suggest that a similar process may exist in neurons. Growth cones of fixed Aplysia bag cell neurons have also been examined via micromechanical measurements. Different growth cone regions yield varying elastic modulus values in the range of 45–225 kPa [35]. Isolated dried and re-hydrated peripheral nerve fibers yield relatively high values, with myelinated and demyelinated fibers both averaging around 800–900 kPa [37].

### 4.2. Living Neurons

Fixation of cells and tissue has been shown to consistently increase the elastic modulus of the sample being measured [9]. As such, measurements on living cells and tissue are often of greater biological significance. While measurement of the elastic modulus of fixed cells may provide useful data on relative stiffness, measuring living cells allows for a better understanding of how the cell might respond to forces within its native environment. For example, chick DRG neurons exhibit preferential outgrowth on substrates with elastic modulus that match the corresponding average elastic modulus of living DRG somas [12,38]. This type of mechanical coupling can only be observed through the measurement of living cells. These types of measurements, however, present additional difficulties over measurements taken on fixed cells. Cell health must be maintained throughout the experiment, which often restricts the timeframe available for the measurements, as well as the maximum forces that can be used. Cell adhesion to the substrate can also be an issue, as cells that are not well attached may slip around under the cantilever, preventing useful measurement. Many techniques are available to circumvent such issues, including the use of heated AFM chambers for measurement [12]. Several studies, described below, have shown that physiologically relevant values for elastic modulus are obtained when cell health and attachment are successfully maintained during the AFM measurements. These techniques are extremely valuable for obtaining information on the neuron structure and elastic properties in response to external stimuli and controlled external forces. The necessary requirement that cells be well adhered to a 2-dimensional substrate, does impose limitations on the technique, such as the capability of measuring the cell structure and responses to controlled external forces *in vivo*, in the native environment of the cell. Despite these limitations, however, the AFM measurements could give valuable insight into how neuronal cells sense external forces and react to mechanical stresses in their natural environment.

#### 4.2.1. Elastic Modulus Measurements

Several types of living neurons and neuronal tissues have been measured with the AFM. Living rat hippocampal slices have been measured by large (25 μm) sphere-tipped AFM cantilevers. It was found that elastic modulus values fall between 50 and 300 Pa, and are dependent on the region of the cell being measured [28]. For example, measurements in CA3 regions (*Cornu Ammonis* area 3 of the hippocampus) yielded stiffer average measurements (230 ± 150 Pa, and 300 ± 180 Pa) than those taken in CA1 regions (170 ± 50 Pa, and 200 ± 130 Pa). Living explants of the fetal rat cortex yield similar values as the hippocampal slices, with an average of 300 ± 25 Pa measured with a 2.5 μm sphere-tipped probe [29].

Elastic modulus values measured via AFM for living neuron somas are available for various cell types. Hippocampal and retinal neurons were measured at various indentation frequencies. An increase in measured elastic modulus value is seen with an increase in indentation frequency. These experiments yielded elastic modulus values around 650 Pa at 30 Hz, and 1590 Pa at 200 Hz [30]. AFM measurements of neuronal cells are typically taken at substantially lower indentation frequencies to limit viscoelastic effects. One study showed that cells respond with an almost ideal elastic response (little to no loading rate dependence) at very low force (30 pN or less), and that at larger forces the measured elastic modulus depends on the loading rate [39]. For larger forces, the dependence of the measured elastic modulus on the loading rate can be minimized by choosing weaker cantilevers, smaller maximum force values, and by minimizing the cantilever loading rates used.

**Figure 4:**
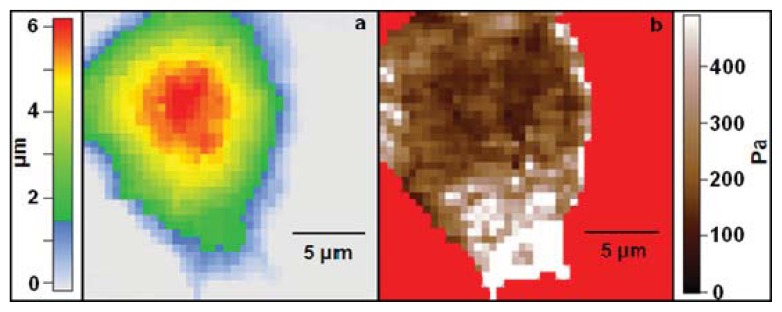
(**a**) Topography of live neuron soma obtained during elastic modulus mapping; (**b**) High resolution elastic modulus map of cell shown in (**a**). The cell displays regions of high elastic modulus localized at the lower end (bright areas in the figure). Images taken by Elise Spedden in Staii lab at Tufts University.

Living DRG, p19 (embryonic mouse teratocarcinoma stem cell) derived, and cortical neurons have all been measured by both individual force curves on varying regions of the cell soma, and by high resolution elastic modulus mapping [12,31,34]. The AFM-based mapping of the cell elastic modulus allows obtaining both height and elastic modulus information with sub-micron resolution. This can be used to measure the distribution of elastic modulus values across a cell (Figure 4).

Live DRG neurons measured by individual force curves are reported to yield elastic modulus values averaging around 60 kPa [34]. Elastic modulus mapping of living DRG’s, however, has yielded averages of around 1 kPa, with individual points on DRG somas ranging between 0.1 and 8 kPa [12]. These values are higher on average than those measured for cells originating from the cortex. This may result from mechanical coupling between cell types and their native growth environment. While cortical and hippocampal neurons must navigate and make connections within tissue measured on the order of hundreds of Pa [28,29], DRG neurons are located along the spinal column connecting the sensory nerves to the central nervous system. This presents a stiffer and more varied environment for growth, as elastic modulus values of the tissue outside the brain typically lie in the kPa range, with spinal cord elastic modulus values falling in the tens of kPa [40]. P19 derived neurons are neuron-type cells differentiated from the mouse p19 stem-cell line. These cells have been measured via sphere-tip AFM indentation yielding average elastic modulus values around 230 Pa [31]. P19 derived neurons have also been measured via AFM elastic modulus mapping, yielding soma average values of around 400 Pa, with individual points on the elastic modulus maps ranging between 100 and 2000 Pa [12]. Indeed, since P19 neurons are typically considered a model system for cortical neurons, they are expected to have similar mechanical properties to those obtained on cortical region neurons [41].

Cortical neuron somas have also been measured by a variety of AFM indentation techniques. Bulk elastic modulus of the cortical neuron soma was measured via repeated oscillations of 45 μm spherical tip probes against the cell body. Rather than computing the elastic modulus using a typical force *vs.* indentation model, such as the commonly applied Hertz model, this study recorded and analyzed repeated oscillations with the aid of a finite element framework simulating experimental testing conditions. The study models cortical soma response to large deformations in a large-strain kinematics framework at various deformation rates. The average value of elastic modulus obtained for the cortical neuron soma was 78 Pa, with a range of 30–200 Pa [32]. These values are consistent with the corresponding values measured through traditional Hertz model analysis of force curves. Cortical neurons measured using individual force *vs.* indentation curves using spherical AFM tips yield soma averages around 100–200 Pa [31]. High resolution elastic modulus mapping of cortical neurons also yields similar values for average soma elastic modulus of around 200 Pa [12]. The observed consistency between different types of measurements, as well as the similarity between the elastic modulus values obtained on individual neurons and the values obtained on bulk tissue measurements of the corresponding cortical region, demonstrate a match between individual cellular elastic modulus values and those for the environment in which they grow [28,29].

AFM topography and elastic modulus measurements have also been performed on living neuronal growth cones. The Aplysia bag cell neuron has a remarkably large and stable growth cone structure, lending itself to detailed topographic and nanomechanical analysis via AFM. AFM topographical imaging allowed one study to locate the P domain lamellipoda, P domain filipodia bundles, T zone ruffles, and C domain of the living growth cone. Individual force *vs.* indentation measurements were taken within each of these regions yielding average elastic modulus values of 16.7, 29.8, 3.5 and 0.5 kPa respectively [35]. It was hypothesized that differences in mechanical stiffness between regions measured, particularly the high values found for the P and T domains, could be important for support and traction generation during growth cone advancement and steering [35]. DRG topography and elastic modulus values have been studied before and after induced sciatic nerve injury, showing regenerative growth post-injury with a lower elastic modulus growth cone (3–13 kPa) than that seen in healthy uninjured growth cones (16–33 kPa). A change in the relative elastic modulus values across measured growth cone regions is also observed [27]. In this study AFM elastic modulus measurements provide unique insight into the post-injury changes of the growth cone, highlighting the differences in elastic modulus of the growth cones between injured and control neurons, along with a drop in actin and increase in βIII-tubulin content [27]. This represents one of several studies that use AFM topography or elastic modulus mapping to track changes to neurons and their structural components over time. Another novel use of this technique was the mapping and tracking of neuronal soma elastic modulus during axonal elongation events. The soma of cortical neurons adjacent to an axon was found to stiffen reversibly during active extension of the axon, with dramatic local stiffening resulting in the average elastic modulus over the whole soma increasing by 30%–180% [12]. AFM provides the unique ability to study such changes in the neuronal cytoskeleton without the need for potentially disruptive fluorescent modification. The same AFM force *vs.* indentation method used to measure living and fixed neurons as described above has also been used to characterize the elastic modulus and topography of various substrates used in neuronal growth experiments. For example, this approach is often used to characterize gels such as polyacrylamide and silk-based surfaces [1,29,33,42].

#### 4.2.2. Combined AFM/Fluorescence Measurements

**Figure 5:**
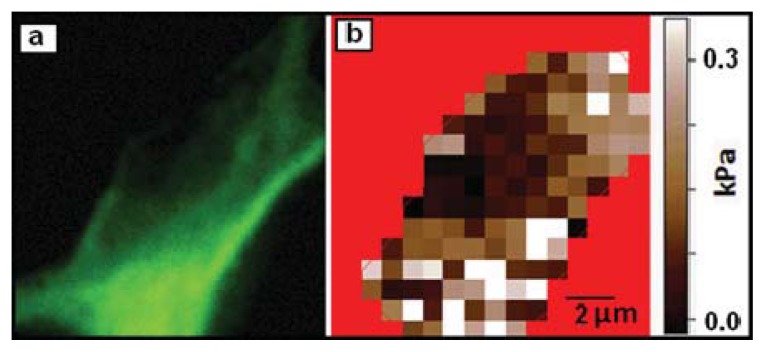
(**a**) Fluorescent image of a living neuron soma stained for tubulin density; (**b**) Elastic modulus map of cell shown in (**a**). The images have the same scale bar shown in (**b**). There is a significant overlap between areas of high actin concentration (high fluorescence intensity) and the regions of high stiffness. Images taken by Elise Spedden in Staii lab at Tufts University.

AFM elastic modulus measurements have been used to monitor live neuronal structures over time. Components within the neuronal soma have been tracked over time using combined AFM mechanical measurements and fluorescence microscopy. For cortical neuron somas maintained at 37 °C, relative elastic modulus values have been used as an indicator for tubulin density. Higher elastic modulus regions typically correlate to dense tubulin aggregations (see Figure 5). Actin density in cortical neurons at 37 °C, however, does not correlate strongly to measured elastic modulus [12]. This technique allows for the tracking of tubulin density within living, unstained cells. The ability to obtain relationships between density of cytoskeletal components and local elastic modulus values at high resolution is currently unique to the AFM. Elastic modulus values can also be tracked for neuronal cells during chemical [12,34] or protein [43] modification. Types of modification explored include Taxol, a stabilizer of microtubules, Nocodazole, an inhibitor of microtubule polymerization, and Blebbistatin, an inhibitor of myosin II contraction of the actin cytoskeleton [12]. This type of measurement offers new insights into the effects that chemical modifiers have on the structure and stiffness of cytoskeletal components of living cells over time. For example, application of a 10 μM dose of Taxol to living neurons has been shown to increase soma elastic modulus by 30% or more, with particular increases in elastic modulus near the tubulin-dense region of the soma close to the axon [12].

## 5. Other Types of AFM-Based Measurements

In addition to the modes discussed in the previous sections, AFM probes can be used in a several other ways to study neurons and their growth. The ability to apply controlled forces using an AFM cantilever can be used to study the mechanosensitive properties of neuron-like cells. By applying controlled mechanical forces via the AFM tip to the growth cones of living NG108-15 and PC12 cells one study determined that mechanical stimulation above a threshold limit resulted in retraction and re-exploration of the stimulated neurite, indicating a mechanosensitive growth-cone response [44]. AFM allowed precise measurement of this stimulation threshold indicating a possible method through which growth cones may sense and react to the stiffness of the growth substrate. AFM can also be used to characterize and/or manipulate the growth environment of neurons. Application of controlled forces to a substrate during AFM scanning can be used to remove surface-bound molecules that inhibit neuronal growth and to promote the attachment of extracellular matrix proteins or adhesion factors in controlled geometries. This technique has been used to position and guide the growth of neuronal cells, and to study the dynamics of axonal extension in controlled geometries [45,46].

Modified AFM probes have been used to track the distribution of AMPA-type glutamate receptors on live hippocampal neurons. Tips were functionalized with antibodies and force *vs.* indentation maps were taken. This technique yields the traditional height and elastic modulus data in addition to strength of binding between the tip and surface during tip retraction. The binding strength was used to monitor antibody-receptor interactions and track receptor density. The results show that receptor density tends to be higher in areas of high local elastic modulus, and that changes in the distribution of the receptors are accompanied by changes in elastic modulus. Similarly, AFM tips coated with nerve growth factors (NGF) have been employed to image surface distribution of NGF receptors across living PC12 cells. This was accomplished through analysis of tip-neuron binding forces during force map acquisition [47]. Finally, we mention that recent experiments have used AFM probes modified with anti-α7 subunit nAChR antibody to measure the ligand-binding properties of α7-containing nicotinic acetylcholine receptors expressed on living neurons. Frequency of adhesion and detachment forces was used to monitor decreases in receptor interaction after exposure to nicotine [48].

## 6. Future Directions

### 6.1. Elastic Modulus and Neuronal Growth

The AFM has proven to be a very effective tool for studying topographic and mechanical features of living and fixed neuronal cells The high-resolution capability of the AFM to obtain physiologically relevant elastic modulus data on living cells may have important future applications. For example, continued exploration of changes in topography and elastic modulus of diseased and dysfunctional neurons may help us gain additional insight into how different neuronal defects contribute to various neurological diseases. As recent studies have shown that certain types of neurons exhibit preferential growth on substrates close to their own average elastic modulus [12,38], expanded data on live neuron elastic modulus under varying conditions might be used to generate better growth substrates for different types of neurons, with applications in regenerative medicine.

### 6.2. Receptor Tracking, Ion Tracking and Membrane Electrostatics

AFM has also been used to track changes in ion concentration along living cells. AFM tips coated with ion-selective polymer have been used to locate areas of high potassium concentrations on the surfaces of MDCK-F1 cells. Adhesion of the modified tips to the surface during force curves was recorded, with higher values of adhesion indicating higher concentrations of potassium ions. These areas of high concentration were used as indicators of the presence of active potassium channels. Relative regional ion channel activity can then be tracked by tracking this adhesion between different curves over time [49]. Such measurements have not yet been performed on neurons; however, similar imaging techniques might be employed to track signaling activity in axons.

This type of work is part of the emerging field of using modified AFM probes to assess binding properties between chemical or antibody modifiers and specific proteins, channels, or receptors along the surface of living neurons [47,48]. Modifications of this type might be used to selectively monitor the activity of such receptors and sites under various chemical and environmental conditions with high spatial resolution, which is currently unavailable by other methods. This type of research is at the forefront of AFM measurement on living cells and is likely to continue as an active field of research in the future.

AFM can also be a powerful tool for analyzing membrane electrostatics [50–52]. Electrostatic parameters of living cell membranes such as surface charge density, surface potential, and transmembrane potential could be mapped via AFM with nanometer-scale resolution [50–52]. Electric Force Microscopy (EFM), for example, has been used to measure surface charge density [50] and membrane dipole potential [51] of lipid bilayers in fluid, and AFM-based electromechanical imaging of collagen fibrils have also been performed [52]. Similar techniques might be adapted to image or control the electrical activity, particularly action potentials, of living neurons cultured on various growth substrates. This technology will be beneficial to neuroscience by allowing researchers to query simple circuits *in vitro* to answer focused questions about neuronal growth and formation of functional connections between neurons.

## 7. Conclusions

During the past few years an increasing number of studies have used the versatility and high spatial resolution of the AFM to probe topographical features and biomechanical properties of neurons. While neuronal cells are some of the weakest in the body, AFM measurement can be sufficiently delicate to preserve the life and health of various types of neuronal cells. This allows for measurement of neuronal structure during growth or regeneration, and the tracking of cytoskeletal components through elastic modulus mapping. The ability to combine AFM measurements with bright field and fluorescence microscopy, as well as to perform these measurements in physiological conditions are valuable to our understanding of the mechanical structure of neuronal cells and how that depends on cytoskeletal components and their dynamics. AFM tip functionalization and novel scanning probe techniques open up new possibilities for tracking non-topographical or mechanical information on living neurons at high resolution.

## Figures and Tables

**Table 1:** Elastic modulus values (*E* values) for neurons measured via AFM.

Fixed/living	Area measured	Animal	Type of neuron	*E* values	Citation
*Fixed*	Soma	Rat	Primary spinal cord neurons	<25–40 kPa	Jiang [33]
*Fixed*	Neurite	Rat	Primary spinal cord neurons	<7.5 kPa	Jiang [33]
*Fixed*	Growth Cone	Aplysia	Bag cell neurons	45–225 kPa	Xiong [35]
Living	Tissue	Rat	Hipocampal slices	52–308 Pa	Elkin [28]
Living	Tissue	Rat	Cortex explants	305 ± 25 Pa	Norman [29]
Living	Soma	Mouse	Hippocampal neurons	480–970 Pa	Lu [30]
Living	Soma	Guinea	Pig Retinal neurons	650–1590 Pa	Lu [30]
Living	Soma	Chick	Dorsal root ganglia neurons	10–140 kPa	Mustata [34]
Living	Soma	Chick	Dorsal root ganglia neurons	1–8 kPa	Spedden [12]
Living	Soma	Mouse	P19-derrived neurons	200–2000 Pa	Spedden [12]
Living	Soma	Mouse	P19-derrived neurons	230 ± 180 Pa	Spedden [31]
Living	Soma	Rat	Cortical neurons	30–200 Pa	Bernick [32]
Living	Soma	Rat	Cortical neurons	100–200 Pa	Spedden [31]
Living	Soma	Rat	Cortical neurons	80–500 Pa	Spedden [12]
Living	Growth Cone	Aplysia	Bag cell neurons	3–40 kPa	Xiong [35]
Living	Growth Cone	Mouse	Dorsal root ganglia neurons	0.4–33 kPa	Martin [27]
